# A Multifaceted Approach to Abdominal Aortic Aneurysm

**DOI:** 10.3400/avd.ra.24-00137

**Published:** 2025-01-21

**Authors:** Katsuyuki Hoshina

**Affiliations:** Department of Vascular Surgery, The University of Tokyo, Tokyo, Japan

**Keywords:** abdominal aortic aneurysm, experimental study: registry: clinical question, hemodynamic, simulation, Japanese Committee for Stentgraft Management

## Abstract

The underlying mechanisms of abdominal aortic aneurysms (AAAs) are not fully understood. Given the multifactorial nature of AAA development and progression, a comprehensive approach is essential. Throughout my academic career, I conducted various studies on AAA. To better understand this mechanism, I initially developed an elastase-infused rat AAA model and applied it to nanoparticle drug delivery systems. While open surgery has traditionally been the standard treatment for AAA, endovascular aneurysm repair (EVAR) has seen significant advancements over the past 25 years. However, insufficient evidence exists regarding this novel treatment, particularly in Japan. To address this issue, we analyzed extensive datasets on EVAR using various registries, including the Japanese Committee for Stent Graft Management. Furthermore, through medical–engineering collaboration, simulation methods were utilized to generate evidence addressing clinical questions encountered in practice.

## 1. Experimental Studies

The elastase-infused rat model is a standard and classical model for abdominal aortic aneurysms (AAAs), replicating many features of human AAA.^1^^)^ Porcine pancreatic elastase infusion directly destroys the AAA wall, forming degradation peptides that attract mononuclear inflammatory cells and trigger matrix metalloproteinase expression, mirroring the pathogenesis of human AAA.^2–4^^)^ Various medications, including anti-inflammatory drugs, have been reported to successfully prevent AAA expansion.^5,6^^)^ I began my AAA-related experimental research using this model from a hemodynamic perspective. Initially, a femoral arteriovenous fistula (AVF) shunt was created to increase aortic flow velocity, while iliac artery ligation was performed to decrease it. Contrary to expectations, AAA expansion was suppressed in the AVF group suggesting that high-flow conditions may stimulate endothelial and smooth muscle cell proliferation.^7^^)^

Simultaneously, we focused on macrophage accumulation around the AAA. A 384-clone DNA microarray analysis of harvested AAA tissue revealed that 29 genes were differentially expressed. Among these, heme oxygenase 1, known to be regulated by hemodynamics, was increasingly localized in infiltrating cells.^8^^)^ We further demonstrated that blood flow regulates macrophage density in the media and adventitia via monocyte chemoattractant protein-1 and granulocyte-macrophage colony-stimulating factor.^9^^)^

Subsequently, I applied this model in the next phase of my research. I delivered the basic fibroblast growth factor (bFGF) gene using in vivo electroporation into medial cells and successfully prevented AAA expansion. Interestingly, gene transfer enhanced medial cell density without increasing the number of endothelial cells or adventitial macrophages.^10^^)^

### Drug delivery system using nanoparticles

Although anti-inflammatory drugs have been primarily used to suppress AAA in animal models, drug toxicity due to large volumes has posed a challenge for clinical applications.^11^^)^ To address this issue, we aimed to develop a specific drug delivery system (DDS) targeting AAA using nanoparticles as drug carriers. We hypothesized that nanoparticles in the bloodstream would accumulate in the AAA wall, where severe disruption and degeneration occurred, and we incorporated rapamycin, known for suppressing experimental AAA via anti-inflammatory mechanisms, into the nanoparticles.^12^^)^ The rapamycin-encapsulated nanoparticle significantly suppressed AAA compared to the sham group treated with rapamycin alone. The accumulation of labeled nanoparticles in the AAA was visualized using an in vivo imaging system and colocalization of nanoparticles with wall cells and macrophages was.^13^^)^

**Figure 1:**
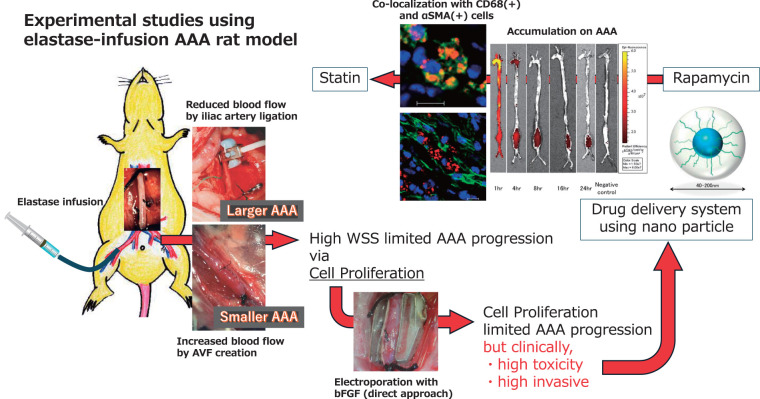
Fig. 1 Experimental studies using elastase-infusion AAA rat model: from hemodynamic model to nanoparticle model. AAA: abdominal aortic aneurysm

Next, we selected statins, known for their pleiotropic effects including anti-inflammatory properties, in addition to their cholesterol-lowering effects, as drugs for targeting AAA suppression. We developed statin-loaded polymeric micelles (pitavastatin), which successfully prevented aneurysm expansion in a dose-dependent manner. Furthermore, the micelle-injected group exhibited reduced macrophage infiltration and matrix metalloproteinase-9 activity in AAA. In vitro cytotoxicity studies using smooth muscle cells revealed that the 50% inhibitory concentration (IC50) of the micelle was approximately 30 times lower than that of free pitavastatin^14^^)^ (**Fig. 1**).

## 2. Registry Studies

### Japanese Committee for Stentgraft Management data

The Japanese Committee for Stentgraft Management (JACSM) was established in 2007 to ensure the safety of endovascular aneurysm repair (EVAR) in Japan. The JACSM registry includes detailed anatomical and clinical data from all patients who underwent stent graft (SG) insertion in the country. Despite the rapid accumulation of data since its establishment, no comprehensive analysis has been performed. One major reason for this is the prolonged and confusing debate regarding ethical approval for the use of registry data, primarily from the perspective of personal information protection. To address this, I developed a scheme facilitating ethical review and approval of registry data through the ethical committee of the University of Tokyo. Subsequently, we analyzed data from 38008 patients who underwent treatment with bifurcated SGs for AAA. The intraoperative and in-hospital mortality rates were 0.08% and 1.07%, respectively. Older age, larger aneurysm diameter, and all types of persistent endoleaks were identified as strong predictors of adverse events, sac dilation, and reintervention. Notably, nearly half (47.6%) of patients violated the anatomical instructions for use (IFU). Furthermore, the sac dilation rate (≥5 mm) at 5 years was 23.3%. Although perioperative outcomes were acceptable, these findings underscore 2 critical safety issues for EVAR operators.^15^^)^

We also analyzed data on thoracic endovascular aortic repair (TEVAR) procedures. Among 22250 TEVAR procedures, 990 patients with type A and 4259 with type B acute dissections were analyzed separately. Our primary focus was on 14235 cases of thoracic aortic aneurysms (TAAs), considering the unique pathogenesis of thoracic aortic conditions. TAAs were classified as elective, urgent (within 24 hours of admission), or emergent (immediately upon admission). Urgency is strongly associated with increased rates of mortality, stroke, and paraplegia, effectively stratifying patients in the long-term survival analysis.

Logistic regression analysis included factors such as the number of debranching bypasses, proximal landing zones (zones 0, 1, 2, or 3), and zone length. A proximal landing zone involving the aortic arch and debranching bypasses were associated with a higher risk of stroke, whereas SG coverage extending over 6 or more zones was associated with a higher risk of paraplegia. These findings provide valuable insights to assist TEVAR operators in developing surgical strategies to mitigate patient risk.^16^^)^ In compliance with the Committee’s requirements, we disclosed the annual EVAR and TEVAR data separately in the 2017 report.^17,18^^)^

### Various studies using JACSM data

The JACSM database has since been made publicly accessible, and I have contributed to several studies as the data manager. In one such study, Seike et al. analyzed 17099 patients under 75 years of age who underwent EVAR, dividing them into 2 groups: those with persistent type II endoleak (p-T2EL) and those without. The study revealed a significant correlation between p-T2EL and late adverse events, including aneurysm sac enlargement, reintervention, rupture, and AAA-related mortality after EVAR.^19^^)^ Ozawa et al. investigated the rupture risk associated with saccular AAA morphology. They analyzed data from 27290 patients who underwent EVAR, examining the distribution of ruptured cases across different AAA diameter ranges. As anticipated, saccular AAAs had smaller diameters than fusiform AAAs in patients with ruptured AAAs. An analysis using the receiver operating characteristic (ROC) curve revealed that the cutoff diameter for predicting rupture was smaller in saccular AAAs than in fusiform AAAs (50.5 mm and 59.5 mm, respectively).^20^^)^ These findings are critical for clinical practice, particularly in determining operative indications.

### Japan AFX Registry

In 2018, the US Food and Drug Administration issued a Class I voluntary recall of the AFX system (Endologix, Inc., Irvine, CA, USA) because of the high rate of type III endoleaks after EVAR.^21,22^^)^ However, no definitive evidence of flaws has been reported in studies utilizing large cohorts with minimal bias. The latest version of the unibody SG, AFX2, was introduced in Japan; however, its data remain unanalyzed. To address this, we established the Japan AFX Registry, recruiting facilities in Japan that used AFX2 in combination with an aortic cuff in at least 5 cases during the initial period. Although the sample size was small (175 patients), detailed data, including CT images, were collected and analyzed.

The midterm outcomes (within 3 years) of using AFX2 with an aortic cuff were acceptable, given the low rate of type III endoleaks. However, 4 cases of sideways displacement were observed, which could lead to future type IIIa endoleaks. Based on these findings, we conclude that when AFX2 is used with an aortic cuff, patients should be closely monitored for potential endograft deformations and subsequent adverse events, including type III endoleaks.^23^^)^

### Cardiac rehabilitation for small AAA study

Nakayama et al. collected data on 1515 small AAA patients prior to surgery from the University of Tokyo Hospital and Sakakibara Heart Institute.^24^^)^ Building on the hemodynamic effects on aortic wall cells described earlier (**Fig. 1**), the study aimed to investigate the beneficial effects of exercise on small AAAs. A carefully tailored cardiac rehabilitation program, designed to prevent excessive blood pressure elevation during exercise, was prescribed to 50 patients with small AAAs. She successfully demonstrated that the program prevented the progression of small AAAs in the rehabilitation group.^24,25^^)^

## 3. Clinical Question and Biomechanical Simulation Studies

Various clinical questions have arisen regarding the treatment of AAA, and we believe that translational research using engineering simulations is necessary to address these questions.

### Morphological change of SGs after implantation

Since the approval of EVAR in Japan, the number of patients undergoing this procedure has increased dramatically. However, cases often arise where the IFU must be evaluated, particularly in patients with severely angulated neck anatomy. In such cases, I observed that implanted SGs sometimes change their morphology over time. To investigate this further, CT images of EVAR cases were analyzed, focusing on post-implantation configurations in severely angulated neck cases. A comparison between the Cook Zenith (Cook Incorporated, Bloomington, IN, USA) and Gore Excluder (W.L. Gore & Associates, Flagstaff, AZ, USA) revealed that the Excluder tended to exhibit recoil, while the Zenith retained its reformed shape or showed further straightening.^26^^)^ Given the drastic morphological changes observed after implantation, I believe that much more careful long-term follow-up is necessary for EVAR patients compared to those undergoing open surgery.

### A case of upward migration of an SG leg

We encountered an 82-year-old female AAA patient with severely angulated neck anatomy who underwent EVAR. The procedure was successfully performed without endoleaks and sac shrinkage reduced the diameter from 73 mm to 47 mm. Ten months later, the patient was admitted to our hospital with acute abdominal pain. A CT scan revealed that the distal edge of the stent-graft limb had migrated upward into the aneurysm sac, resulting in a type 1b endoleak and impending rupture.

### Medical–engineering collaboration and simulation

**Figure 2:**
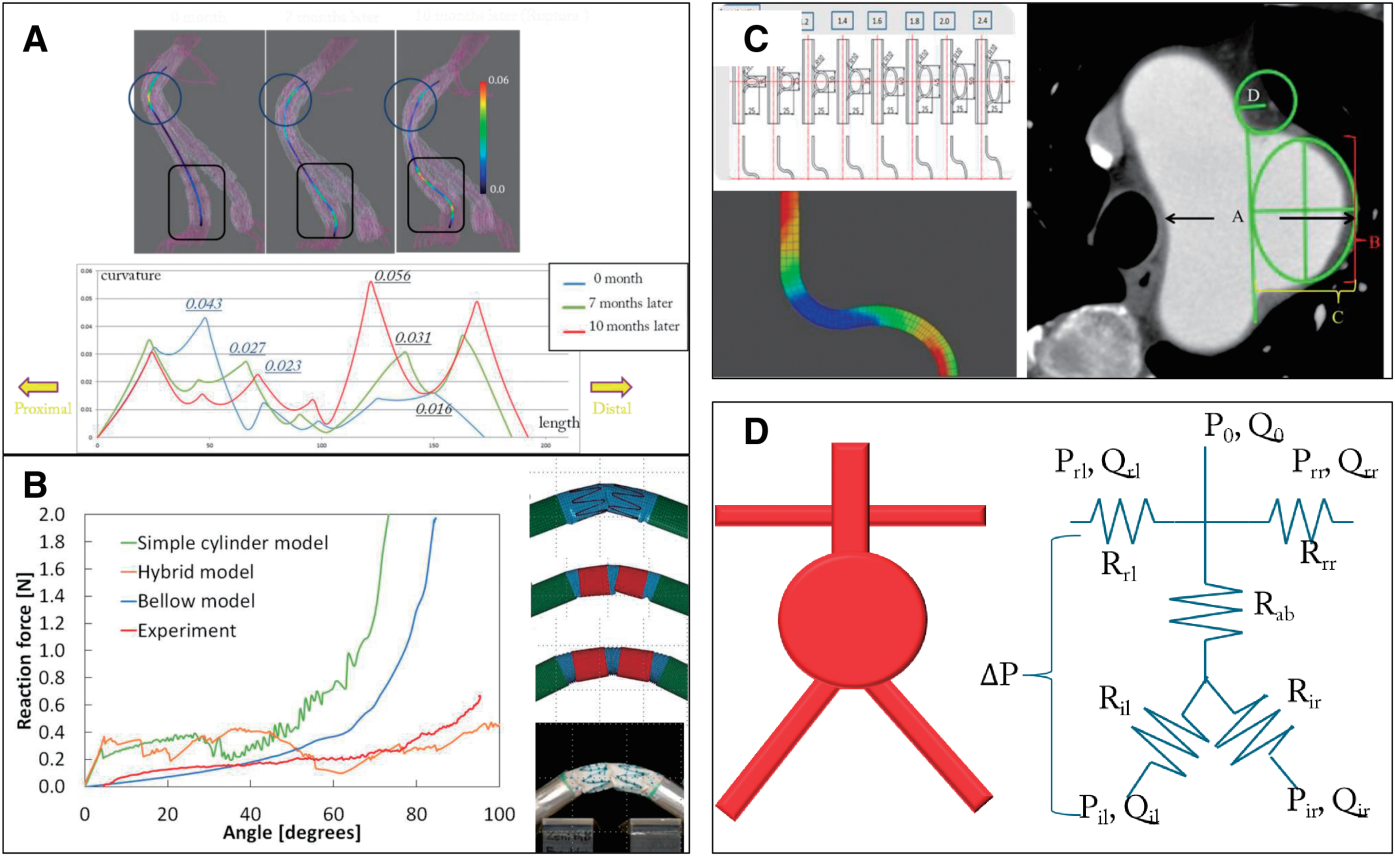
Fig. 2 Biomechanical simulation studies. (**A**) Visualization and quantification of stent graft curvature. (**B**) The device models with ideal bending behavior. (**C**) Definition of “saccular” aneurysm. (**D**) A zero-dimensional electronic circuit model for evaluating renal artery flow change after AAA surgery. AAA: abdominal aortic aneurysm

This case highlights the need for a theoretical (numerical) evaluation of device morphology and collaboration between the medical and engineering fields. In 2010, we initiated the “Aortic Aneurysm Simulating Meeting” in collaboration with the Oshima Lab at the Interfaculty Initiative in Information Studies, The University of Tokyo, and the Yamamoto Lab at the Department of Engineering Science and Mechanics, Shibaura Institute of Technology. We developed a modeling system called V-Modeler, an effective tool for constructing 3D models of both the aortic lumen and the SG. The system evaluates vascular and SG geometries using parameters such as length, curvature, torsion, angle of the tangent vector, and migrated length. The results were visualized and quantified, revealing that the strong peak of curvature shifted from the proximal to the distal end, while torsion gradually diminished over time^27^^)^ (**Fig. 2A**). We applied this method clinically to the Excluder and Zenith SGs.^28^^)^ The study demonstrated the behavior of SGs after implantation, providing numerical values for SG length and curvature. Surprisingly, the rate of distal migration was much higher than expected (19%), compared to a much lower migration rate reported in the registry data (2.8%).^15,17^^)^

We further investigated the mechanical properties of each device and tested the bending using tactile samples to examine the relationship between the angle and reaction force. We explored the device models with ideal bending behavior (**Fig. 2B**).

### What is “saccular” AAA?

The average AAA diameter reported in our registry was approximately 51 mm, smaller than those reported in previous studies from the United States and Europe. This discrepancy may be due to the inclusion of a significant number of patients with saccular AAA in our dataset. While saccular AAAs are thought to carry a higher rupture risk compared to fusiform AAAs, we felt it was necessary to define “saccular” to perform EVAR with appropriate surgical indications. To address this issue, we constructed a simple aortic aneurysm model with 2 components: a tube representing the aorta and an ellipse representing the bulging aneurysm. By varying 3 parameters (the vertical and horizontal diameters of the ellipse and the fillet radius), we used structural analysis with the finite element method to visualize the distribution of maximum principal stress (MPS) in the aortic wall and identified areas of high stress. We applied the theoretical results to imaging data from patients with TAAs, in whom aneurysm expansion rates were monitored. The maximum MPS increased significantly in areas where the aspect ratio (vertical/horizontal) was <1, indicating that “horizontally elongated” ellipses should be defined as “saccular” aneurysms. The aneurysm expansion rate in patients with TAAs conforming to these parameters was significantly higher^29^^)^ (**Fig. 2C**). We collected cases of ruptured AAAs and matched them with non-ruptured AAAs, applying the saccular method to both groups. Unlike saccular forms, ruptured AAAs were characterized by a small fillet radius while the protrusion rate appeared less significant. Clinically, we concluded that local aortic deterioration (small fillet radius) might be a more significant contributing factor for rupture than aneurysm protrusion (large aspect ratio).^30^^)^ We applied this methodology to the CT images of AAAs and compared the subjective clinical judgment of “saccular” morphology with the objective assessment derived from mechanical structural analysis. In the analysis of real CT data, the clinical judgment of vascular surgeons differed from the results obtained through mechanical structural analysis using the application model. Based on this, we concluded that the saccular morphology may theoretically be rare in AAAs.^31^^)^

### Theoretical renal blood flow increases after AAA repair

Another clinical observation after open surgery or EVAR, especially for juxtarenal or pararenal AAAs, is the occasional temporal improvement in renal function immediately post-operation.^32^^)^ We initially thought that postoperative transfusion might contribute to this improvement. However, we hypothesized that other factors, such as a reduction in the infrarenal blood flow area following surgery, might contribute. To explore this hypothesis, we calculated the theoretical reduction in blood flow area after AAA repair. A zero-dimensional electronic circuit model and a diagram of blood flow distribution were developed by assigning resistance values to the aorta, bilateral iliac arteries, renal arteries, and aneurysm. Using the radius and length of each anatomical parameter to calculate theoretical values, we found that renal blood flow increased by 13.4% after surgery^33^^)^ (**Fig. 2D**). Although these results are only theoretical data, a correlation was observed between aneurysm volume regression and improvements in estimated glomerular filtration rate (eGFR). Based on this theory, improvements in postoperative renal function may be more pronounced in patients with larger AAA.

## 4. Blind Men and an Elephant

**Figure 3:**
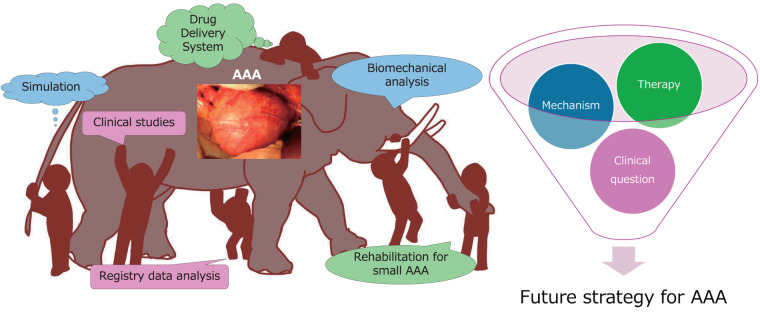
Fig. 3 Blind men and an abdominal aortic aneurysm.

Although this is akin to a blind man touching only parts of an elephant and attempting to describe the whole, we hope that each of these complementary approaches to understanding AAA mechanisms contributes to the development of future treatment strategies (**Fig. 3**).

## 5. Conclusion

The development and dilation of AAA involve multiple factors. The evidence presented here represents pieces of the puzzle in this scenario. Further translational research is essential and should be shared with the next generation. We aspire to reduce the number of deaths caused by ruptured AAAs and to improve the safety and efficacy of AAA treatment.

## Declarations

### Disclosure statement

None.
